# Rituximab as a sustainable high-efficacy solution for multiple Sclerosis in Brazil

**DOI:** 10.1055/s-0046-1823678

**Published:** 2026-06-25

**Authors:** Cintia Ramari, Giordani Rodrigues dos Passos, Jefferson Becker

**Affiliations:** 1 Pontifícia Universidade Católica do Rio Grande do Sul, Faculdade de Medicina, São Lucas Hospital, Porto Alegre RS, Brazil.; 2 Hasselt University, Faculty of Rehabilitation Sciences, REVAL Rehabilitation Research Center, Hasselt, Belgium.

Multiple sclerosis (MS) remains the leading cause of non-traumatic neurological disability among young adults worldwide, with an estimated 2.8 million people affected globally and approximately 35,000 in Brazil. 1 The therapeutic landscape has undergone a paradigm shift, moving away from traditional escalation strategies toward the early initiation of high-efficacy treatments to mitigate long-term disability and prevent conversion to progressive disease. 2 3 However, in low- and middle -income countries such as Brazil, access to these therapies remains limited by financial constraints and regulatory barriers. 3 4

In this issue of *Arquivos de Neuro-Psiquiatria* , Simon-Nogueira et al. provide important real-world evidence from a Brazilian tertiary center demonstrating that rituximab, despite its off-label status, represents a potent and feasible therapeutic option within the Brazilian Unified Health System (SUS). Their study offers a timely contribution by addressing the gap between an ever-growing therapeutic arsenal and real-world access to newly approved drugs, a challenge that is particularly pronounced in resource-constrained healthcare systems.

Rituximab, a chimeric monoclonal antibody targeting CD20+ B cells, was the first B-cell–depleting therapy to demonstrate clinical efficacy in MS, although its formal development in this indication was later superseded by newer anti-CD20 agents such as ocrelizumab, ofatumumab, and ublituximab. 2 5 6 Despite this, due to its much lower annual cost, rituximab has emerged as a pragmatic substitute in routine care in many countries, driven not only by biological plausibility but also by necessity. 3 4 For instance, in Sweden, it has become one of the most frequently prescribed disease-modifying therapies. 6 7

The biological rationale supporting this substitution is well established. Early clinical trials with rituximab demonstrated substantial reductions in inflammatory disease activity. In the OLYMPUS trial, 8 although the primary endpoint in primary progressive MS was not met, subgroup analyses suggested benefits in younger patients and those with active inflammatory lesions, supporting the role of B-cell depletion as a central mechanism in MS pathophysiology. These findings ultimately paved the way for the development of ocrelizumab, which confirmed the efficacy of anti-CD20 therapy across MS phenotypes.

The Brazilian experience reported by Simon-Nogueira et al. mirrors international observations and reinforces the growing body of evidence supporting rituximab as an effective high-efficacy therapy in clinical practice. Importantly, the Brazilian context adds a critical dimension to this discussion. While ocrelizumab and ofatumumab have demonstrated robust efficacy in large phase III trials – namely the OPERA I and II studies, the ORATORIO study, and the ASCLEPIOS I and II studies, 2 which showed significant reductions in relapse rates and disability progression against placebo or active comparators – and secured marketing approval in Brazil, their incorporation into the SUS has not been approved, due to financial concerns. Differently from Sweden, however, neither has rituximab been incorporated as an off-label alternative. As a result, access to anti-CD20 agents remains arguably one of the main unmet needs in the treatment of MS in the Brazilian public health system.

Within this framework, the results presented by Simon-Nogueira et al. are both consistent and clinically meaningful. Their cohort demonstrated a marked reduction in annualized relapse rate, with 90.9% of patients remaining relapse-free following rituximab initiation. These findings are remarkably aligned with data from the Swedish RIFUND-MS trial, 6 which reported similarly high rates of relapse freedom, despite important differences in study populations. While RIFUND-MS evaluated an early, treatment-naïve cohort with low disability, the Brazilian study reflects a more complex and advanced patient population, with longer disease duration, higher disability scores, and prior exposure to multiple therapies.

Beyond efficacy and cost, the safety profile of rituximab is well established through decades of use in oncology and rheumatology, and accumulating evidence in MS has further supported its favorable safety profile in long-term clinical practice. 5 6 Although concerns regarding infections and hypogammaglobulinemia remain—particularly with long-term use—these risks are generally manageable with appropriate monitoring strategies. 9 Notably, the primary reason for treatment discontinuation reported in the study by Simon-Nogueira et al. was not safety or lack of efficacy, but discontinued access to the medication. This finding highlights a critical paradox: a therapy that is clinically effective and economically advantageous may still be inaccessible due to regulatory and logistical barriers.

The economic implications of these findings are equally relevant. In Brazil, only a small proportion of patients within the public system receive high-efficacy treatments as first-line therapy, largely due to financial constraints. 3 If ocrelizumab and ofatumumab are deemed unaffordable by the Brazilian public health system policy makers, then rituximab might well represent a lower-cost alternative to finally provide access to anti-CD20 therapy. 3 7 This has been corroborated by international data demonstrating lower overall healthcare costs and favorable clinical outcomes associated with rituximab use in MS. 4 7

The inclusion of rituximab in the World Health Organization (WHO) List of Essential Medicines for MS, along with only two other MS drugs – glatiramer acetate and oral cladribine – further reinforces its relevance, particularly in resource-limited settings. 7 In Latin America, where disparities in healthcare infrastructure and access remain substantial, rituximab has the potential to reduce the therapeutic gap and enable earlier access to high-efficacy treatment. 4 The latest Brazilian Consensus for the Treatment of Multiple Sclerosis, 10 published in 2018 by the Brazilian Academy of Neurology and the Brazilian Committee on Treatment and Research in Multiple Sclerosis, also recognizes rituximab as an acceptable alternative for individuals with highly active MS. This reality underscores a broader issue in global MS care: therapeutic decisions are often shaped as much by access as by evidence.

From a methodological perspective, the findings of Simon-Nogueira et al. should be interpreted within the context of the study design. As a retrospective, observational study, it is inherently subject to selection bias and lacks a comparator group. The relatively small sample size limits the ability to perform detailed subgroup analyses, and the inclusion of a substantial proportion of patients with progressive disease may reduce the interpretability of annualized relapse rate as an outcome measure. Additionally, real-world challenges such as inconsistent MRI monitoring and variability in dosing regimens reflect systemic constraints rather than methodological shortcomings. The single-center nature of this study, particularly the fact it was conducted in a tertiary care, highly specialized MS center, prevents extrapolation of its findings for the entirety of neurology centers and MS-treating professionals across in Brazil, whose familiarity with – and access to – risk-mitigation strategies pertinent to rituximab varies significantly. These limitations are not unique to this study but are representative of the broader challenges faced in generating real-world evidence in Latin America. 1

Despite these constraints, this work represents a pioneering contribution to regional MS literature. It provides one of the first structured datasets evaluating rituximab use within the Brazilian public healthcare system and offers valuable insights into treatment effectiveness under real-world conditions.

In conclusion, the study by Simon-Nogueira et al. offers important and timely insights into the role of rituximab as a high-efficacy, affordable, and sustainable treatment option for MS in Brazil. While acknowledging its methodological limitations, the study underscores the importance of aligning therapeutic strategies with healthcare realities. In settings where access to approved high-cost therapies remains limited, rituximab represents not only a viable alternative but also a critical tool for promoting equity in MS care. More broadly, this work marks an important step toward building a Latin American evidence base that reflects the unique challenges and opportunities of the region.
